# Caregiver Experiences and Resource Gaps Following Pediatric Traumatic Brain Injury: A Qualitative Analysis

**DOI:** 10.3390/bs16101848

**Published:** 2026-10-09

**Authors:** Jingzhen Yang, Archana Kaur, McKenna Fowler, Christine H. Koterba, Sarah Case, Krista K. Wheeler, Ping Zhang, Jaclyn Caccese

**Affiliations:** 1 Center for Injury Research and Policy, Abigail Wexner Research Institute, Nationwide Children’s Hospital, Columbus, OH 43205, USA; archana.kaur@nationwidechildrens.org (A.K.); krista.wheeler@nationwidechildrens.org (K.K.W.); 2 Department of Pediatrics, The Ohio State University, Columbus, OH 43210, USA; christine.koterba@nationwidechildrens.org; 3 School of Health and Rehabilitation Sciences, The Ohio State University, Columbus, OH 43210, USA; mckenna.fowler@osumc.edu (M.F.); jaclyn.caccese@osumc.edu (J.C.); 4 Department of Neuropsychology, Nationwide Children’s Hospital, Columbus, OH 43205, USA; 5 Community Advisory Board, Metro Cafe at MetroHealth, Cleveland, OH 44109, USA; sarahldcase@gmail.com; 6 Department of Computer Science and Engineering, The Ohio State University, Columbus, OH 43210, USA; zhang.10631@osu.edu; 7 Department of Biomedical Informatics, The Ohio State University, Columbus, OH 43210, USA

**Keywords:** pediatric TBI, rehabilitation navigation, care coordination, transition to home, family adjustment

## Abstract

This qualitative study explored caregivers’ experiences navigating their child’s recovery following pediatric traumatic brain injury (TBI) and identified barriers and facilitators to accessing healthcare, educational, and community resources after hospital discharge. Semi-structured virtual interviews were conducted with caregivers of children with a history of TBI from Ohio and other U.S. states. Interviews were transcribed verbatim and analyzed using thematic analysis. Among the 13 caregivers, 69% were female, and 92% were White. Three themes emerged: (1) caregivers as primary navigators of fragmented systems; (2) the impact of recovery on family life and well-being; and (3) the need for personalized, longitudinal support throughout recovery. Caregivers described assuming primary responsibility for coordinating services across healthcare, rehabilitation, insurance, education, and community systems with limited guidance during care transitions. They also reported emotional exhaustion, social isolation, financial strain, and the ongoing demands of advocating for their children’s changing needs. Although supportive clinicians, care coordinators, and community organizations facilitated recovery, families continued to face challenges accessing information, coordinating services, and navigating available resources. Findings underscore the need for longitudinal, family-centered models of care that extend beyond current standards. Centralized, technology-enabled navigation may reduce caregiver burden by improving information access, care coordination, and personalized support throughout recovery.

## 1. Introduction

Traumatic brain injury (TBI) is a leading cause of morbidity and long-term disability among children and adolescents (Baticulon et al., 2022; Haarbauer-Krupa et al., 2018; Langlois et al., 2005). Advances in acute care have improved survival, resulting in more children living with the long-term effects of TBI and often facing prolonged recovery marked by evolving physical, cognitive, behavioral, and emotional challenges (Blackwell & Grell, 2023; Goh et al., 2021; Keenan et al., 2021). Caregivers play a key role throughout recovery by coordinating care, advocating for services, supporting rehabilitation, and facilitating reintegration into home, school, and community life in a way they never did before the TBI (Azman et al., 2020; Brown et al., 2013).

Despite their central role, caregivers frequently report feeling unprepared to navigate the complex and fragmented systems they encounter after their child’s hospital discharge (Aitken et al., 2009; Holloway et al., 2019; Kirk et al., 2015; Minney et al., 2019). Recovery after pediatric TBI often requires coordination across healthcare, rehabilitation, insurance, educational, and community systems, yet communication and support across these settings are frequently inconsistent or poorly integrated (Ciccia et al., 2021; Palusak et al., 2022). Although families receive substantial information in the hospital, many struggle to identify appropriate resources and guidance after hospital discharge as new challenges emerge over time (Jones et al., 2018; Lundine et al., 2026; Scheuer et al., 2026). These difficulties may contribute to delayed access to services, increased caregiver burden, and ongoing uncertainty throughout recovery (Haarbauer-Krupa et al., 2017).

Prior research has documented caregiver stress, unmet needs, and challenges following pediatric TBI (Brenner et al., 2021; Dahl et al., 2024; Fuentes et al., 2018), yet less is known about how families navigate recovery across different phases of care and how support needs evolve over time. Understanding caregivers’ lived experiences across rehabilitation, transition to home, and long-term recovery is critical for developing family-centered systems that improve care coordination and longitudinal support beyond the acute phase.

This qualitative study explored caregivers’ experiences navigating recovery following pediatric TBI in Ohio and other U.S. states and identified barriers and facilitators to accessing healthcare, educational, and community resources after hospital discharge. Findings from this study can inform the development of centralized, adaptive, and technology-enabled navigation supports to help families access understandable and timely resources across the recovery trajectory.

## 2. Materials and Methods

### 2.1. Study Design

We conducted a qualitative study using semi-structured interviews with 13 caregivers of children with a history of TBI. A qualitative approach was selected to gain timely, practice-relevant insights into families’ experiences navigating healthcare services, rehabilitation, and recovery resources following pediatric TBI (Hamilton & Finley, 2019). All participants provided verbal informed consent before participation. Study design and reporting were guided by the Consolidated Criteria for Reporting Qualitative Research (COREQ) checklist (Tong et al., 2007).

Research team members with expertise in qualitative methods, pediatric injury research, and family-centered care were trained to conduct interviews and qualitative data analysis. One interviewer was also a TBI survivor, bringing lived experience that supported empathetic engagement with participants (Patton, 2014).

### 2.2. Participants and Recruitment

Parents or primary caregivers of children with a history of TBI were recruited through clinical partners, community organizations, and parent support networks in Ohio and other U.S. states. Recruitment materials were distributed through the Ohio Brain Injury Association listserv, ResearchMatch, StudySearch, and word-of-mouth referrals. Eligible participants were adults who identified as a primary caregiver of a child who sustained a TBI before age 18. Interested parents contacted the research team through email or a QR code included on study flyers. Research staff screened participants for eligibility and scheduled virtual interviews. Purposive sampling was used to capture variation in caregivers’ experiences across rehabilitation, transition to home, and long-term recovery. Recruitment continued until thematic saturation was reached, defined as the point at which additional interviews yielded no substantively new themes (Riger & Sigurvinsdottir, 2016). A final sample included 13 parent participants; because all served as their child’s primary caregiver following TBI, we use the term caregiver throughout the manuscript.

### 2.3. Interview Guide Development

The semi-structured interview guide was developed through an iterative, stakeholder-informed process informed by prior literature on pediatric TBI recovery, caregiver experiences, and transitions in care (Diener et al., 2022; Jenkin et al., 2022; Lundine et al., 2019; Maggard et al., 2023; Scheuer et al., 2026; Wade et al., 2023). Caregivers of children with TBI contributed to the interview guide development by identifying key challenges, information needs, and recovery experiences. The process began with discussions between the research team and a caregiver of a child with TBI, whose insights, together with findings from the literature, informed the initial draft of the interview guide. The draft was subsequently reviewed by researchers with expertise in pediatric TBI and qualitative methods, as well as clinicians who care for children with TBI. The revised guide was pilot tested with another caregiver of a child with TBI to assess its clarity, relevance, and flow. Feedback from the pilot interview informed additional revisions to improve wording and ensure sensitivity to caregiver experiences. Final revisions were guided by input from a community advisory board that included clinicians, caregiver stakeholders, and community partners.

### 2.4. Data Collection

Semi-structured interviews were conducted virtually via Microsoft Teams by two trained research team members (AK and MF) and lasted approximately 60 min. Interviews explored caregivers’ experiences and burden across the recovery trajectory following pediatric TBI, including rehabilitation, transition to home, long-term adaptation, and school reintegration, and perspectives on centralized or technology-enabled navigation support and care coordination. For this study, analyses focused on three areas: (1) navigating systems and care transitions (e.g., “Can you describe the transition from the hospital to rehabilitation and what that looked like for you and your family?” and “What challenges did you experience navigating insurance, scheduling, or coordinating care?”); (2) effects of recovery on caregivers and family functioning (e.g., “What was the hardest part for you and your family about transitioning your child back home?” and “What adjustments did your family need to make during recovery?”); and (3) support needs across recovery (e.g., “What resources or supports were available to help you?” “What additional support would have made a difference?” and “How did changes in your child’s recovery over time shape the support your family needed?”). All interviews were video-recorded, professionally transcribed verbatim, and de-identified prior to analysis. Interviewers also completed field notes after each interview to document contextual observations, reflections, and preliminary analytic insights, which were used to support data interpretation.

### 2.5. Data Analysis

Transcripts were analyzed using thematic analysis (Kogen, 2024; Riger & Sigurvinsdottir, 2016). A preliminary codebook was developed based on the interview guide and initial transcript review. Two transcripts were independently coded to refine coding categories and incorporate emergent concepts. Intercoder reliability was established between two coders (AK and MF) to ensure consistency and resolve discrepancies. After finalizing the codebook, the two coders independently conducted line-by-line coding of all 13 transcripts using ATLAS.ti 25 (Bariar et al., 2026). Regular team meetings were held to review coding consistency, resolve discrepancies, and refine emerging themes.

Following coding, transcripts were summarized using a structured template to capture key experiences, barriers, facilitators, and representative quotations. The research team then grouped related codes into broader categories and synthesized them into overarching themes and subthemes reflecting caregivers’ experiences (Crabtree & Miller, 2023). Several strategies were used to enhance analytic rigor, including team-based coding, iterative comparison across transcripts, regular consensus meetings, reflexive discussions, and an audit trail documenting coding decisions and theme development (Patton, 2014).

## 3. Findings

### 3.1. Participant Characteristics

Of the 13 parents interviewed, most were female (69%), White (92%), and held a four-year college degree or higher (84%). The average parent age was 45 years (Table 1). Children were between 3 and 17 years old at the time of injury, with a mean age of 9.8 years. Most children with TBI were male (77%). At the time of the interview, the average time since injury was 5½ years, ranging from 1 to 25 years.

### 3.2. Themes and Subthemes

Three themes emerged regarding caregivers’ experiences navigating rehabilitation and recovery following pediatric TBI: (1) caregivers as primary navigators of fragmented systems; (2) the impact of recovery on family life and well-being; and (3) the need for personalized, longitudinal support throughout recovery. Within each theme, subthemes captured the barriers and facilitators that families identified as influencing recovery (Table 2).

#### 3.2.1. Theme 1. Caregivers as Primary Navigators of Fragmented Systems

This theme captured caregivers’ experiences navigating fragmented systems and coordinating care following their child’s TBI. Limited guidance and disconnected services often required families to assume primary responsibility for managing recovery. Four subthemes were identified: (1) limited guidance and care coordination across transitions, (2) advocacy as an essential but burdensome caregiver role, (3) structural barriers to accessing services, and (4) coordinated professional support facilitated navigation.

##### Limited Guidance and Care Coordination Across Transitions

Caregivers described transitions between care settings as stressful and poorly coordinated, often leaving families responsible for navigating healthcare, rehabilitation, insurance, and educational systems with little guidance regarding rehabilitation options, outpatient services, or follow-up care. One caregiver reflected, “*Nobody tells you that you have a choice*” (P09), while another expressed frustration that “*hospitals did not provide the information families expected during recovery planning*” (P11). Additionally, another shared, “*Someone managing his care and translating for a layman was missing*” (P07).

As children transitioned home, caregivers frequently assumed responsibility for coordinating services, scheduling appointments, and navigating insurance requirements. One caregiver described hiring outside assistance to manage insurance claims and denials because caregiving demands had become overwhelming, while another reported “*receiving limited guidance regarding how to maximize outpatient therapy services covered by insurance*” (P12). Challenges also extended into the educational system. One caregiver described attempting to return her child to a “*normal school year*,” only to discover that ongoing behavioral challenges required individualized tutoring. Another noted that she was responsible for “*communicating with schools and coordinating accommodations*” following her child’s injury (P06).

##### Advocacy as an Essential but Burdensome Caregiver Role

Caregivers frequently described advocacy as a central responsibility, often monitoring care, seeking information, and pursuing services when they felt their child’s needs were not adequately recognized or addressed. Several caregivers reported requesting referrals, seeking additional medical opinions, or advocating for changes in treatment plans. One caregiver explained that she “*sought additional medical opinions when providers did not respond to ongoing concerns*” (P01), and another recalled, “*We had to like really advocate for all the services”* (P10). Caregivers also emphasized the importance of remaining actively engaged in clinical decision-making, with one caregiver describing the need to be “*actively seeking information during rounds and treatment discussions to remain informed about medical decisions*” (P07).

Advocacy demands became increasingly complicated, particularly when cognitive, behavioral, and social difficulties were overlooked because children appeared outwardly well. One caregiver recalled a teacher stating that her child appeared “normal” despite continued post-injury challenges: “*You know, and then you say that to me, it was terrible. So, but it did get better after that…we’ve just been very, very strong advocates for X*” (P08). As a result, caregivers often found themselves repeatedly explaining their child’s injury and related needs while advocating for accommodation and services across multiple settings.

##### Structural Barriers to Accessing Services

Caregivers described numerous barriers that limited access to rehabilitation and recovery services following pediatric TBI. Geographic distance, transportation challenges, provider shortages, and insurance restrictions frequently complicated efforts to obtain needed care. One explained, “*the nearest pediatric rehabilitation centers were located hours away*” (P01), while another described independently researching long-distance transportation options because “…*no social work assistance was available*” (P02).

Caregivers often reported difficulties identifying pediatric specialists, obtaining referrals, and securing insurance-covered therapies. One caregiver described hiring a private occupational therapist because “*insurance-covered services were insufficient*” (P03). Families living in rural communities faced additional challenges locating speech, occupational, and rehabilitation services close to home. One caregiver noted, “*There isn’t one in this area, so you’ll either have to travel 2 hours South or an 1 ½ North to get to find a pediatric facility.*” (P01).

##### Coordinated Professional Support Facilitated Navigation

Despite challenges navigating fragmented systems and coordinating care, caregivers consistently identified coordinated professional support as a key facilitator of recovery. Social workers, case managers, care coordinators, and healthcare providers helped families navigate transitions, access services, and better understand complex medical information. One caregiver explained that “*Case managers were helpful when I told them exactly what needed to happen*” (P03), while another described rehabilitation teams managing “*the logistics*” of transition planning, including assistance with temporary housing accommodations.

Families also valued support that reduced the burden of coordinating care after discharge. One caregiver explained that “*care coordinators helped families obtain needed services and supports*” (P06). Caregivers emphasized the importance of providers who communicated clearly, explained rehabilitation goals, and supported shared decision-making. One caregiver recalled relying on “*advice from nurses when making difficult treatment decisions*” (P07), while others valued multidisciplinary meetings that provided opportunities to discuss recovery goals, Medicaid options, and future planning. For instance, “*There was a social worker… I remember him… trying to be helpful. The main advice was like, get on these Medicaid waiver programs…*” (P13).

#### 3.2.2. Theme 2. The Impact of Recovery on Family Life and Well-Being

This theme captured the emotional, social, practical, and financial impact of pediatric TBI on families. Caregivers described significant challenges adapting to their child’s recovery while managing changes in family life, caregiving responsibilities, and personal well-being. Three subthemes emerged: (1) managing complex care at home; (2) emotional, social, and financial strain; and (3) family, community, and emotional support promoted adaptation.

##### Managing Complex Care at Home

Many caregivers felt unprepared to manage their child’s evolving medical, behavioral, and safety needs after discharge. The transition to home often brought significant anxiety as families assumed responsibility for monitoring symptoms, managing behavioral changes, ensuring safety, and making day-to-day care decisions with limited support. One caregiver described, “*I remember the nurse coming and saying, here’s your discharge papers. And my husband was getting the car, I said, well, what are we supposed to follow up? Like, do we, who do we follow up with? Do we come back here?*” (P01). Another caregiver explained that transition to home without inpatient nursing support felt “really scary” because the family became responsible for managing their child’s complex medical needs and applying the skills learned in the hospital to the home setting. Several caregivers similarly described aspects of their inpatient rehabilitation experiences that left them feeling unprepared for discharge. One recalled, “*therapists entering simultaneously to complete assessments, creating an overwhelming experience*” (P07).

##### Emotional, Social, and Financial Strain

Caring for a child with TBI affected family relationships, emotional well-being, employment, and financial stability acutely and long after the initial injury. Caregivers frequently described exhaustion, competing responsibilities, and disruptions that affected the entire family. One caregiver described the return home as a “*huge transition*” because siblings struggled with receiving less attention and adjusting to changes in family routines. Another summarized the experience as “*sheer exhaustion*” because caregivers received little support while managing extensive caregiving demands.

Many caregivers also described feelings of isolation and emotional burden. One caregiver reflected that families “*feel so lost and isolated during recovery*” (P01), while another described developing “*tunnel vision*” and spending “*all day, every day*” focused on her child’s recovery (P09).

Financial strain further compounded the emotional toll of caring for a child with a brain injury. Some caregivers were unable to return to work following their child’s injury, resulting in substantial income loss and ongoing stress. One caregiver described “*struggling financially after losing income while continuing to pay for therapies and services*” (P09).

##### Family, Community, and Emotional Support Promoted Adaptation

Despite the challenges they faced, caregivers identified several factors that supported their family’s adaptation and resilience following their child’s TBI. Strong support networks were essential to family adaptation and resilience. Emotional, practical, and social support from family members, friends, employers, community organizations, and healthcare professionals helped families navigate hospitalization, rehabilitation, and long-term recovery. One caregiver recalled that “*a close friend traveled to accompany her child during emergency transport*” (P01), while another described how “*the whole community stepped up*,” organizing fundraisers and providing ongoing support throughout recovery. Families also valued connections with other caregivers who had experienced pediatric TBI through informal networks and social media groups.

Healthcare professionals were another important source of support. Caregivers described clinicians, therapists, counselors, and social workers as trusted advocates who provided reassurance, guidance, and emotional support during periods of uncertainty. Counseling and psychological services were viewed as particularly beneficial for helping families cope with stress and adjust to life after injury. One caregiver characterized therapy as “*the biggest help*” in managing emotional stress.

#### 3.2.3. Theme 3. The Need for Personalized, Longitudinal Support Throughout Recovery

This theme reflected caregivers’ perspectives on the support needed to navigate pediatric TBI recovery over time. Families emphasized the importance of accessible, personalized, and longitudinal support that adapts to children’s changing developmental, educational, and recovery needs and helps them access appropriate resources throughout the recovery trajectory. Three subthemes emerged: (1) recovery needs evolved over time, (2) resources were available but difficult to find and use, and (3) individualized and adaptive support facilitated recovery.

##### Recovery Needs Evolved over Time

Caregivers emphasized that pediatric TBI recovery was highly individualized, non-linear, and often extended years beyond the initial injury. Many described new cognitive, behavioral, social, and educational challenges emerging months or years after injury as children progressed through different developmental stages. Some caregivers expressed frustration that these evolving difficulties were often unrecognized by educators, healthcare providers, or others because children often appeared outwardly well. One caregiver explained that her child was considered “*fine*” despite ongoing support needs related to TBI. Caregivers also reported concerns that providers did not always recognize individual recovery trajectories, which could affect access to ongoing services and support.

##### Resources Were Available but Difficult to Find and Use

Caregivers consistently reported that resources existed but were difficult to locate, interpret, and apply. Families frequently described independently searching for services, coordinating information across providers, and determining which resources were most relevant to their child’s changing needs. One caregiver described creating “*a big spreadsheet*” to organize rehabilitation information and manage recovery-related tasks. Another caregiver explained that resources were available, “*but you had to go hunt for it*”, reflecting the substantial effort required to identify and access appropriate support.

Although families valued educational materials and rehabilitation information, many felt overwhelmed by the amount of information provided during hospitalization and desired more accessible guidance over time. One caregiver described a rehabilitation class that explained recovery phases and reassured families that challenging behaviors were part of healing, while another noted that “*Each of those transition points [are] incredibly difficult and it’s scary and you don’t know what you resources you need, and at each point there’s no one to help you*” (P03). Several caregivers cited how the lack of centralized navigation support makes it hard to access and organize resources. One participant proposed that “*a virtual social worker*” could help families navigate services after discharge.

##### Individualized and Adaptive Support Facilitated Recovery

Despite challenges throughout recovery, caregivers emphasized the value of individualized support tailored to their child’s needs, interests, developmental stage, and recovery goals. One caregiver described therapists “*incorporating her child’s interests into therapy sessions to improve engagement*” (P05), while another recalled providers extending rehabilitation services because they recognized the child’s potential for continued recovery. Families also appreciated rehabilitation approaches that included caregiver training and preparation for managing recovery at home.

Many caregivers developed their own adaptive strategies to address ongoing challenges and promote independence. One caregiver described creating a sleeping area next to her child’s bed to monitor nighttime wandering and ensure safety, while another family hired a personal trainer to help rebuild strength and independence during recovery: “*He wanted a personal trainer [because] he had he lost [muscle]. I mean he was like skin and bones. That was the one thing he asked for was to workout and try to get stronger*” (P09). Families also valued equipment, home modifications, and practical caregiving training that supported day-to-day functioning.

## 4. Discussion

This qualitative study explored caregivers’ experiences navigating recovery following pediatric TBI. Findings suggest that the challenges families face after pediatric TBI stem not only from the injury itself but also from the burden of navigating disconnected healthcare, education, insurance, and community systems with limited guidance. Although caregivers identified supportive clinicians, care coordinators, and community networks as important facilitators, they consistently described gaps in coordination and longitudinal support as children’s needs evolved over time. Many of these challenges, including caregiving burden, unmet informational and support needs, and difficulties navigating care, are shared by caregivers of children with other chronic conditions (Thomas et al., 2023); however, they may be particularly salient in pediatric TBI because of its often less visible and evolving effects. Children may appear physically recovered while continuing to experience cognitive, behavioral, emotional, social, or educational difficulties that become more apparent as developmental and academic demands increase, complicating caregivers’ efforts to obtain appropriate recognition, accommodations, and services. Our findings reinforce prior literature documenting unmet family needs after pediatric TBI (Aitken et al., 2009; Brown et al., 2013; Ciccia et al., 2021; Holloway et al., 2019; Kirk et al., 2015; Minney et al., 2019), while extending this work by highlighting the dynamic nature of caregiving and resource-navigation needs across recovery and development and the ongoing challenge of identifying, accessing, and coordinating the right support across multiple systems at the right time (Narad et al., 2019; Nichols et al., 2026; Wade et al., 2006).

A key finding was that caregivers frequently felt unprepared to manage emerging challenges after hospital discharge. Families described uncertainty surrounding symptoms, behavioral changes, safety concerns, school issues, and service needs, often with limited access to timely guidance outside of scheduled clinical encounters. Consistent with prior research (Brenner et al., 2021; Brown et al., 2013; Scheuer et al., 2026), caregivers frequently relied on internet searches, social media groups, personal networks, and self-developed organizational systems to fill information gaps. Importantly, caregivers suggested that the challenge was not always the absence of resources but the inability to access relevant, trustworthy information when needed. These findings support the development of centralized navigation systems that provide responsive, just-in-time guidance throughout recovery rather than relying solely on information delivered during hospitalization (Erlick et al., 2021; Jenkin et al., 2022).

Caregivers consistently described taking on the role of care coordinators across healthcare, rehabilitation, insurance, education, and community systems. They often assumed primary responsibility for arranging services, communicating among providers, navigating insurance requirements, advocating for accommodation, and coordinating school support. These findings align with previous reports of fragmented service delivery following pediatric TBI (Brenner et al., 2021; Brown et al., 2013; Scheuer et al., 2026) and extend this literature by illustrating how coordination challenges persist across multiple systems and recovery phases (Hagen et al., 2025). Families’ experiences suggest that future navigation interventions should move beyond resource directories and instead facilitate communication and coordination across systems. Models that integrate healthcare providers, care coordinators, rehabilitation specialists, school personnel, and community resources may help reduce caregiver burden and improve continuity of care (Palusak et al., 2022; Shook et al., 2022). Because these experiences are shaped by the healthcare, insurance, educational, and community service systems in which families navigate care, the findings should be interpreted within the U.S. context and may not directly generalize to countries with different service structures.

Caregivers emphasized that pediatric TBI recovery is highly individualized and evolves over time. New cognitive, behavioral, emotional, and educational challenges frequently emerged months or years after injury, particularly during developmental transitions and school progression. Consistent with previous research (Baticulon et al., 2022; Blackwell & Grell, 2023; Goh et al., 2021), caregivers described difficulties obtaining recognition and support for challenges that were not immediately visible, highlighting the dynamic nature of pediatric TBI recovery. Families also consistently valued individualized rehabilitation approaches that incorporated children’s strengths, interests, developmental needs, and recovery goals (Shen et al., 2023). These findings suggest that support systems should be adaptive rather than static, with resources and guidance tailored to children’s changing needs across developmental stages and recovery phases.

### 4.1. Implications for Clinical Practice and System Design

Our findings suggest that pediatric TBI recovery should be viewed as a longitudinal, family-centered process rather than a discrete episode of care. Current discharge practices may not adequately address the informational, emotional, educational, and coordination needs that emerge long after hospitalization. Healthcare systems should prioritize coordinated transition planning, longitudinal follow-up, and family-centered navigation support beginning during hospitalization and extending throughout recovery (Jenkin et al., 2022; Palusak et al., 2022). The findings further support the development of centralized, technology-enabled navigation systems designed specifically for families of children with TBI. Such systems could provide personalized resource recommendations, facilitate communication among healthcare, educational, and community providers, offer timely guidance as new challenges arise, and help caregivers track services, appointments, and recovery goals. By reducing the burden of locating information and coordinating care across fragmented systems, these approaches may improve access to services, reduce caregiver burden, and better support children and families throughout recovery.

### 4.2. Limitations

This study has several limitations. The relatively small sample, which consisted predominantly of White and college-educated caregivers, may limit the transferability of the findings, particularly to racially and ethnically diverse, socioeconomically disadvantaged, and other underserved populations who may experience different or greater barriers to accessing and coordinating services. Future studies should include larger and more diverse caregiver populations to determine whether additional themes or perspectives emerge. Participants were recruited primarily through support networks and community organizations in the United States, which may have introduced selection bias. The U.S.-based sample may also limit transferability to countries with different healthcare and service systems. Additionally, retrospective interviews may be subject to recall bias, particularly given the variation in time since injury (1–25 years), although only one caregiver reported on an injury occurring more than 10 years before the interview (25 years). Although a child’s TBI may represent a significant life event for caregivers, the accuracy and detail of recollections may diminish or change over time. The experiences reported may also vary by injury severity, geographic location, healthcare access, age at injury, and time since injury. Finally, because the study focused on caregiver perspectives, the findings likely do not fully capture the experiences of children, siblings, educators, or healthcare providers.

## 5. Conclusions

Caregivers of children with TBI frequently became the primary navigators of fragmented systems while managing the emotional, practical, and financial demands of recovery. Although supportive clinicians, care coordinators, and community networks helped families adapt, caregivers consistently described gaps in guidance, coordination, and long-term support as their children’s needs evolved over time. These findings highlight the importance of viewing pediatric TBI recovery as a longitudinal, family-centered process rather than a discrete episode of care. Future research should examine these findings in larger and more diverse populations and develop and evaluate family-centered navigation approaches that provide timely information, facilitate coordination across healthcare, rehabilitation, education, and community systems, and adapt to families’ changing needs throughout recovery. Cross-national research is also needed to determine which navigation challenges are shared across settings and which reflect specific healthcare and social-service systems. Such research can inform scalable and context-responsive approaches to reducing caregiver burden, improving access to services, and supporting children with TBI and their families.

## Figures and Tables

**Table 1:** Characteristics of caregiver participants and their children with TBI.

Participant	Caregiver Sex	Caregiver Age, y	Child Age at Injury, y	Child Sex	Years Since Child’s Injury
P01	Female	≥60	3	Female	>10
P02	Female	30–40	13	Male	1–5
P03	Female	50–60	16	Male	1–5
P04	Male	Unknown	12	Male	1–5
P05	Male	30–40	7	Female	1–5
P06	Female	30–40	7	Female	1–5
P07	Female	≥60	16	Male	6–10
P08	Female	40–50	16	Male	1–5
P09	Female	50–60	10	Male	1–5
P10	Female	20–30	17	Male	1–5
P11	Male	40–50	3	Male	6–10
P12	Male	40–50	5	Male	6–10
P13	Female	40–50	3	Male	6–10

Note: TBI = traumatic brain injury.

**Table 2:** Themes, subthemes, operational definitions, and representative quotations.

Subtheme	Operational Definition	Representative Quotations
Theme 1. Caregivers as primary navigators of fragmented systems		
1.1. Limited guidance and care coordination across transitions	Limited guidance and poor coordination across care settings required caregivers to independently manage recovery.	“Each of those transition points is incredibly difficult and it’s scary… and at each point there’s no one to help you.” (P03)
		“We really didn’t have a clear idea of what was going on.” (P13)
1.2. Advocacy as an essential but burdensome caregiver role	Caregivers advocated for their child’s needs across healthcare, education, and support systems.	“I had to be the advocate with no training.” (P07)
		“I played the main role. I had to communicate with the school. I had to communicate with her doctors and try to get them to, you know, come into the appointments and staff at the school. And it was just hard.” (P06)
1.3. Structural barriers to accessing services	Systemic barriers limited access to rehabilitation and support services.	“We [were] traveling an hour and a half to Marshall, the trauma center for a while when we first got in for follow-ups.” (P03)
		“There was issue like they had a Ronald McDonald House, but it was even unclear if we could even go there.” (P04)
1.4. Coordinated professional support facilitated navigation	Professional support improved care coordination and system navigation.	“So, they had like this class that they brought us to and explained like the different steps of what to expect and the fit.” (P02)
		“We found out by an organization called Team Luke Hope for Minds… that’s where we got a lot of our ideas on how to get the Medicaid waiver.” (P11)
Theme 2. The impact of recovery on family life and well-being		
2.1. Managing complex care at home	Caregivers managed complex medical, behavioral, and rehabilitation needs at home.	“That verbal disconnect there… that was a rough hurdle.” (P08)
		“The very first part for us, or at least for me, was the not having the nurses there… keeping an eye medically on her. That was hard at first, really scary.” (P05)
		“I don’t know how other people handle this kind of situation; it’s like a complete wild west” (P11)
2.2. Emotional, social, and financial strain	Recovery placed substantial emotional, social, and financial burdens on families.	“And…, my other daughter, felt a lot of guilt too, like survivor guilt, because she wasn’t really hurt.” (P01)
		“That just went through me because I cried myself to sleep at night because of just how different things were.” (P08)
		“The occupational therapist is not covered by insurance, so I ended up not using the nursing hours that I was granted by insurance.” (P03)
2.3. Family, community, and emotional support promoted adaptation	Social, emotional, and practical support promoted family adaptation and resilience.	“Connecting with other parents who’d been in the same situation was incredibly helpful.” (P03)
		“There was this massive amount of support… social workers, nurses, doctors, just everybody.” (P05)
		“They gave us resources, they gave us the therapist, they gave us someone up there that stayed with us the whole time.” (P07)
Theme 3. The need for personalized, longitudinal support throughout recovery		
3.1. Recovery needs evolved over time	Support needs changed as children’s recovery progressed over time.	“They don’t really understand traumatic brain injury… people think because they look normal that everything is OK.” (P09)
		“Most people did not realize there was any deficits…Most people, I don’t think, really notice. In fact, people would come up to me and say I’m so glad everything worked out. And I just didn’t have an answer.” (P01)
3.2. Resources were available but difficult to find and use	Caregivers struggled to identify and access relevant resources and services.	“We could have used someone to better sit down with us periodically because as I said, you can’t even take it all in, but to kind of sort of run through the realm of possibilities.” (P13)
		“And there was some information out there about that, but I would have appreciated more about dealing with the emotional challenges.” (P11)
		“It’s very hard to find someone that knows anything about brain injury” (P09)
3.3. Individualized and adaptive support facilitated recovery	Personalized and adaptive support promoted recovery and independence.	“The child life specialist came in and they like put on some audio books and left him with like some video game podcasts…things that he could lightly listen to for a little bit of stimulation without looking at screens.” (P10)
		“I organized physical therapy because we happened to know people there and they were an advocate for him because he had been teaching rock climbing.” (P07)
		“We were needing an ADA bathroom. Like we didn’t have a handicap accessible bathroom. We have one bathroom for five people. As I said, we live in a rural area.” (P09)

## Data Availability

The datasets used and/or analyzed during the current study are available from the corresponding author upon reasonable request. The datasets are not publicly available due to privacy restrictions. The corresponding author had full access to all the data in the study and takes responsibility for the integrity of the data and the accuracy of the data analysis.

## Abbreviation

The following abbreviation is used in this manuscript:

TBI	Traumatic Brain Injury
